# New bioassay cage methodology for *in vitro* studies on *Varroa destructor* and *Apis mellifera*

**DOI:** 10.1371/journal.pone.0250594

**Published:** 2021-04-26

**Authors:** Rassol Bahreini, Medhat Nasr, Cassandra Docherty, David Feindel, Samantha Muirhead, Olivia de Herdt

**Affiliations:** Plant and Bee Health Surveillance Section, Alberta Agriculture and Forestry, Edmonton, AB, Canada; University of Crete, GREECE

## Abstract

*Varroa destructor* Anderson and Trueman, is an ectoparasitic mite of honey bees, *Apis mellifera* L., that has been considered a major cause of colony losses. Synthetic miticides have been developed and registered to manage this ectoparasite, however, resistance to registered pyrethroid and organophosphate Varroacides have already been reported in Canada. To test toxicity of miticides, current contact-based bioassay methods are designed to evaluate mites and bees separately, however, these methods are unlikely to give an accurate depiction of how miticides interact at the colony level. Therefore, the objective of this study was to develop a bioassay cage for testing the toxicity of miticides on honey bees and *Varroa* mites simultaneously using amitraz as a reference chemical. A 800 mL polypropylene plastic cage holding 100–150 bees was designed and officially named “Apiarium”. A comparison of the effects of three subsequent dilutions of amitraz was conducted on: *Varroa* mites placed in glass vials, honey bees in glass Mason jars, and *Varroa-*infested bees in Apiariums. Our results indicated cumulative *Varroa* mortality was dose-dependent in the Apiarium after 4 h and 24 h assessments. Apiarium and glass vial treatments at 24 h also had high mite mortality and a positive polynomial regression between *Varroa* mortality and amitraz dose rates. Moreover, chemical application in the Apiarium was less toxic for bees compared to the Mason jar method. Considering these results, the Apiarium bioassay provides a simple, cheap and reliable method for simultaneous chemical screening on *V*. *destructor* and *A*. *mellifera*. Furthermore, as mites and bees are tested together, the Apiarium simulates a colony-like environment that provides a necessary bridge between laboratory bioassay testing and full field experimentation. The versatility of the Apiarium allows researchers to test a multitude of different honey bee bioassay experiments including miticide screening, delivery methods for chemical products, or development of new mite resistance-testing methodology.

## Introduction

*Varroa destructor* Anderson & Trueman, is an important ectoparasitic mite affecting honey bees, *Apis mellifera* L., and is a major challenge in managed colonies. *Varroa* feed on immature and mature bees causing a decrease in newly-emerged bee weight, reduced hypopharyngeal gland activity, a reduction in protein storage capacity, and a shortened lifespan [1–4]. In addition, *Varroa* are primary agents for transmitting pathogenic viruses to honey bees [5, 6]. The impact of *Varroa* on colonies varies with environment, climate, management practices, colony parasites and pathogen loads, and the strain of bee [1, 7, 8]. Four synthetic miticides have been registered in Canada to control *Varroa* since their introduction in the 1990s [9]: Apivar® (amitraz, Veto Pharma, France), Apistan® (*tau*-fluvalinate, Vita Europe, UK), Bayvarol® (flumethrin, Elanco, Canada), and CheckMite+^TM^ (coumaphos, Elanco, Canada). According to the annual report from the Canadian Association of Professional Apiculturists [10], beekeepers in Canada rarely rely on Apistan®, Bayvarol® or CheckMite+^TM^ for *Varroa* control, as there are concerns and reports from producers of low efficacy for these Varroacides. Resistance to Apistan® and CheckMite+^TM^ was documented in Canada in 2001 and 2002, respectively [11], however, there are no published reports regarding resistance of *Varroa* to Apivar® or Bayvarol® [11, 12]. Currently, there is a significant concern that continuous reliance on Apivar® as a major control option for *Varroa* will eventually lead to the development of resistance, leaving the Canadian beekeeping industry without effective synthetic *Varroa* control options. Information on emerging amitraz-resistant *Varroa* populations across Canada is limited; however, resistance to amitraz has been documented in the United States, Argentina and Mexico [13–15]. Without alternatives for management of *Varroa*, a miticide with a new mode of action is urgently required to safeguard the health of honey bees and the beekeeping industry. Screening new compounds on bees and mites together would expedite the process; however, a reliable laboratory method resembling a colony environment to test efficacy is required.

Historically, laboratory bioassays have evaluated the effects of biotic and abiotic factors on honey bees using cages made from plexiglass, wood, or plastic material. Some investigations have also used Petri dishes [16, 17], plastic caps [18], polycarbonate [19], or cardboard boxes [20]. For instance, the grooming behavior of *A*. *mellifera* against *Varroa*, and how *Nosema* spp. infection influences the level of grooming were examined in hording wooden cages [21, 22]. In addition, the effect of protein feed on bee physiology and behavior has been evaluated using Plexiglass cages [23–25]. Huang et al. [26] compared the cage design and feeding systems in eleven cages with different shapes, materials, and feeding regimes. They found three different cages with 20 mL plastic syringe feeders, with the addition of protein to feeding regimes, had the highest honey bee survivorship. More recently, the effect of miticides on honey bees was evaluated in 60 mL glass Mason jars [27]. In this method, a group of newly-emerged worker bees were contact-exposed to chemicals covering the inner surface of jars.

Bioassay techniques used for testing chemicals on mites and bees, more specifically, have mainly focused on determining the level of resistance of *Varroa* to currently available miticides. In these bioassays, techniques such as chemically coating the inner surface of vials or jars [27–29], small capsules covered with paraffin [30], chemically impregnated Parafilm wax [31] or plastic containers [13] have been used. For instance, Rinkevich [13] evaluated amitraz resistance in mite populations of some beekeeping operations in the USA using a 946 mL polypropylene plastic container. In his study, a group of approximately 300 *Varroa*-infested bees were exposed to a piece of Apivar® strip (4 x 4 cm) for a 3 h period. According to Rinkevich [13], 100% *Varroa* mortality was evaluated in amitraz-susceptible mites in comparison to <80% in resistant populations. Only a few studies exist that are aimed at testing new synthetic chemicals for *Varroa* control [27, 29, 32]. Ali et al. [32] treated honey bee colonies with tebufenpyrad and found a high efficacy for this compound. At the same time, tebufenpyrad resulted in a reduced brood area, adult population, and an increased incidence of queen failure. Vandervalk [29] conducted one of the first laboratory trials to screen the efficacy of commercial synthetic miticides, not registered in Canada, for the treatment of *Varroa* including: Apollo® (50% clofentezine), Forbid® (24% spiromesifen), Floramite® (22.6% bifenazate), and Shuttle® (15.8% acequinocyl). The experiment used 20 mL borosilicate glass vials where the inner surface was coated with different concentrations of miticides. Results indicated that *Varroa* had a higher susceptibility to Shuttle® followed by Apollo®, Forbid® and Floramite®. In a more recent study, Bahreini et al. [27] also used the vial test to screen chemicals against mites and a Mason jar test for bee toxicity. Results showed that fenazaquin and etoxazole had high efficacy for *Varroa* control. From these laboratory studies [27, 29], high efficacy miticides need to be screened more thoroughly under field or colony-like conditions.

Laboratory bioassays are critical for the initial screening of new miticides for efficacy against *Varroa*, nevertheless, some limitations exist that need to be addressed before scaling up to field experiments. In some cases, miticides are tested exclusively on mites to determine the lethal dose or effective dilution of an active ingredient [27, 33]. Honey bees are not only affected by miticides but are an integral part of how miticides spread throughout the colony and how they are delivered to *Varroa*. In addition, when determining miticide efficacy, continuous exposure of *Varroa* to miticides in contact-bioassays (glass vial technique) may produce partial results. Glass vials do not accurately represent a colony environment where the chance of encountering the miticide is much lower. The delivery method (e.g. strip, gel, vapor, etc.) for a new miticide is an important component of testing efficacy. In most cases, the delivery method for application is reserved for field experiments, which often requires a large investment of finances, time, and resources. Initial testing of application methods in the laboratory before moving to field trials could reduce these research constraints.

In light of all the limitations described for previous laboratory bioassays, a new polypropylene plastic cage for testing miticides on *Varroa* was designed in 2017 to address some of these concerns [34]. Developing a bioassay cage that is able to simulate a colony-like environment to test both honey bees and *Varroa* mites simultaneously, and provide reliable results, is a critical step in screening new miticides. This method could expedite the process of finding new chemicals for treating *Varroa* and contribute to a more robust integrated pest management system for beekeepers. Using amitraz as a reference chemical, the objectives of this study were to develop a new bioassay method for laboratory evaluation of miticide efficacy, and to compare the newly developed cage against two commonly used contact-method bioassays. If amitraz performed well in the new method, it would likely provide a better indication of how future chemicals would perform in a colony environment before executing a full-scale field trial.

## Materials and methods

This study was undertaken in the summer of 2017, at the Crop Diversification Centre North (CDCN), Edmonton, Alberta, Canada (53.54°N, 113.49°W). All bees used in this bioassay were provided from non-migratory European honey bee (*A*. *mellifera*) colonies. The experimental bee colonies were managed using standard management practices [35] to reduce variabilities among *Varroa* mite and honey bee populations. The experimental colonies were managed in standard Langstroth boxes with two brood chambers and headed by Kona queens (Hawaii, USA). They were fed syrup for wintering and treated according to label directions, if needed, with Apivar® or oxalic acid sublimation for *Varroa* control, and Fumagillin-B® (Medivet Pharmaceutical Ltd., High River, AB, Canada) for *Nosema* spp. treatment. Bee colonies were wrapped in western winter wraps and wintered outdoors [36]. Prior to the experiment, two groups of colonies were established. Following Bahreini et al. [27], the first set of colonies had high *Varroa* mite infestation levels (>3%), and received two drone frames in early July to further increase their mite populations. The second set of colonies were kept in a separate yard with no mites or low mite levels (<1%) and considered healthy colonies. To ascertain the initial arithmetic mean abundance [37] of mites in each set of colonies, the alcohol wash method as described by Bahreini et al., [27] was used.

### New plastic cage description

**Fig 1:**
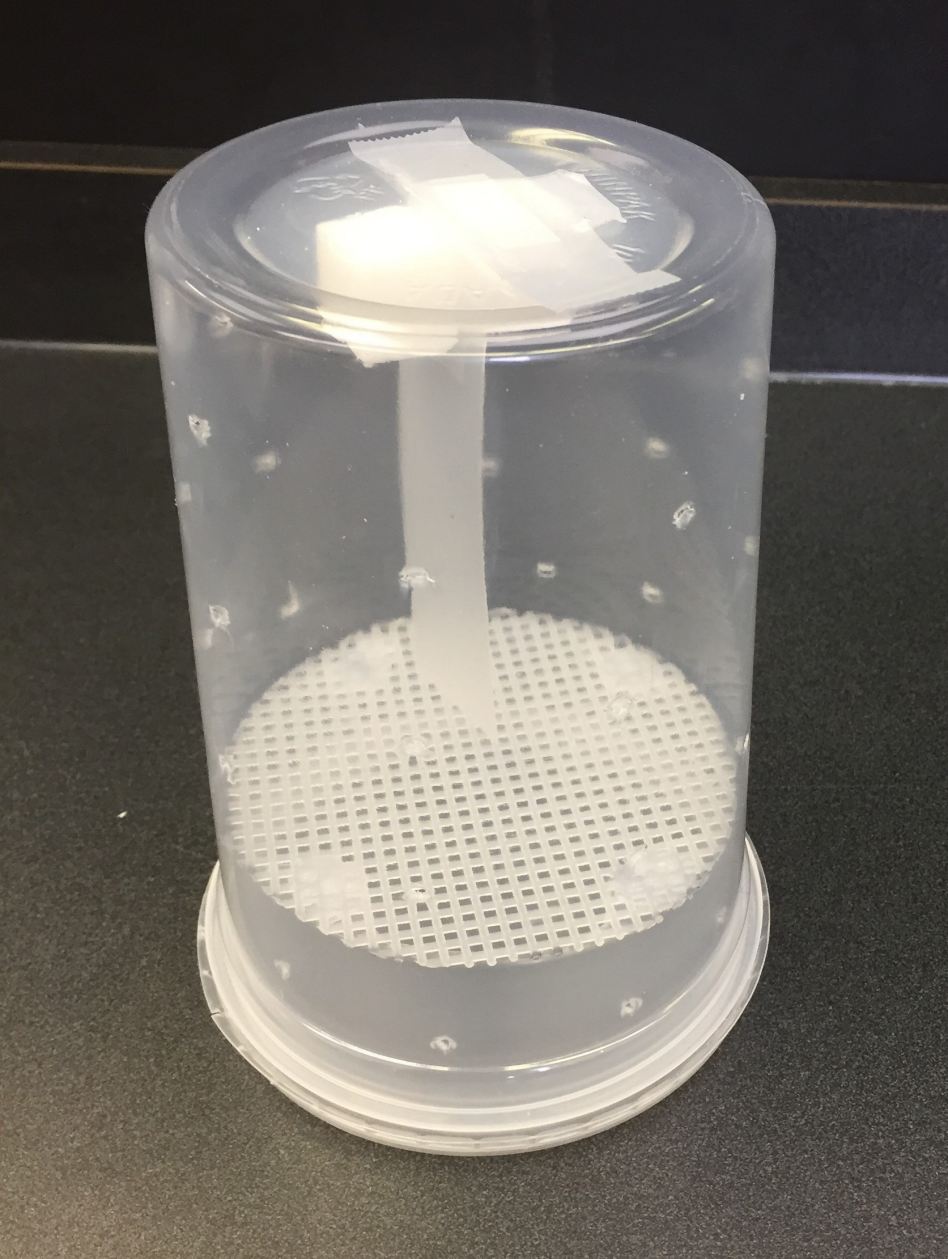
Prototype of Apiarium. Each Apiarium included one small 125 mL (bottom part) and one large 1000 mL (top part) disposable plastic container. Four small tabs were cut on the bottom of 125 mL container to adhere a plastic mesh screen (#7). On the bottom of the 1000 mL container, a 5 cm slit was cut to insert a strip covered with chemicals. Holes were made on the body of both containers for air ventilation. Two sugar cubes (bee food) were secured on either side of the slit using hot glue. To set up the Apiarium, the lid on the 125 mL container was attached, and the 1000 mL container was placed on top of the 125 mL container. The Apiarium’s final volume was 800 mL.

The main structure of the cage includes one 125 mL polypropylene plastic container (top diameter 11.5 cm x bottom diameter 10 cm x height 4.5 cm) that fits into a 1000 mL polypropylene plastic container (top diameter 11.5 cm x bottom diameter 9 cm x height 15 cm) with a lid. All container pieces were obtained from Plastipak Industries Inc., SK, Canada. The bottom of the 125 mL container was removed except for four 1 cm x 1.5 cm tabs around the edge of the container. The tabs were used as a platform to adhere a plastic canvas mesh screen (10.16 cm diameter, #7, Michaels Canada) that was cut to the size of the bottom of the 125 mL container. The mesh screen allowed dead mites to fall through to the bottom of the cage for counting while also preventing bees from escaping when the lid was removed. A 5 cm x 2 mm incision was cut into the bottom of the 1000 mL plastic container using a razor blade and holes were punctured along the side using a dissecting needle to allow for ventilation. A piece of plastic strip (2.54 cm x 11.43 cm, Recycled Binding Cover, Staples Canada) covered with an active ingredient or solvent was placed through the incision to the inside of the container and taped in place. Two sugar cubes were glued to the bottom of the container adjacent to the strip. The 125 mL container with mesh bottom was placed inside the 1000 mL container with the lid attached and the structure was inverted to complete the design. The total volume of the final main chamber in the container was 800 mL. The plastic cage will hereafter be referred to as an “Apiarium” (Fig 1).

#### Mite and bee collection for vial and Mason jar assays

Collection of live mites, newly-emerged bees, and pupae was done according to methods described by Bahreini et al. [27]. To collect mature female mites, highly *Varroa*-infested bees were placed in a secure container and shaken on an orbital digital shaker (VWR, Canada) at 300 rpm for 10 minutes, while being exposed to CO_2_. To ensure the mites used in the study were susceptible to amitraz, resistance to Apivar® was evaluated on the experimental colonies before mite collection, using an adapted version of the Pettis method [38] as described in Bahreini et al. [27]. Mites in this experiment were collected from colonies prior to oxalic acid or Apivar® treatments. In order to provide newly-emerged worker bees for the Mason jar assay, capped brood frames from healthy bee colonies (<1% *Varroa* mite infestation) were confined in a screened wooden cage (50 cm x 26 cm x 7 cm) and incubated at 33±1°C and 60±5% RH in the dark until bees emerged from their cells. Purple-eyed pupae were also prepared from healthy bee colonies for the vial test following the same method.

#### *Varroa*-infested bee collection for the Apiarium assay

All bees used in the Apiarium were homogeneous and well mixed from the experimental colonies. Mix-aged bees were collected from the brood frames of highly *Varroa*-infested colonies (11.73 ± 0.96% mean abundance). Collection was done by shaking bees from brood combs into a Rubbermaid container (10 L). In the field, one quarter cup (60 mL) of bees was transferred directly into the Apiariums using a ladle. Each Apiarium had an average of 135 bees (134.63 ± 7.61) and 15 mites (15.44 ± 1.32).

### Amitraz as a reference chemical

Amitraz was used as a reference chemical to determine if the Apiarium could be a suitable method to screen promising chemicals for *Varroa* treatment in the future. Amitraz is a primary treatment option in Canada and was highly effective on the local mite population at the time of this study.

### Glass vial assay for mites

All vial methods were adopted from Bahreini et al. [27]. Borosilicate scintillation glass vials (20 mL; Wheaton, Fisher, Canada) (n = 20) were treated with 0.5 mL of a subsequent dilution 10,000 mgL^-1^, 1,000 mgL^-1^ or 100 mgL^-1^ of the active ingredient Amitraz Pestanal® (amitraz 99.99%, Sigma-Aldrich, Canada). Each vial was treated with 3.91 mg, 0.391 mg or 0.0319 mg of amitraz. Acetone (density 784 gmL^-1^, 99.9%, Sigma-Aldrich Canada) was used as the solvent. With four replicates each, the dilutions were pipetted onto the inner surface of the vial. Two additional sets of replicates were added: acetone only (solvent control), and no treatment (negative control). As described in Bahreini et al. [27], each treated vial was rotated on a hot dog roller, with the heating system turned off, until the solvent completely evaporated and the residue of the compound evenly coated the inner side-surface of the vial. On the day of the experiment, ten mites were collected and introduced to the treated vials using a fine-tipped paintbrush. New labelled paintbrushes were used for each dilution. Prepared vials were incubated at 33±1°C and 60±5% RH in the dark and mite mortality was determined after 4 h exposure to amitraz. The surviving mites in each vial were transferred into a 2 mL centrifuge tube (Fisher, Canada) and fed one freshly-collected purple-eyed bee pupa. After an additional 20 h, mite mortality was counted (24 h post-treatment). Mites that demonstrated no response to contact with a fine-tipped paintbrush (i.e. lack of appendage movement) were considered dead. The mites and vials were disposed of appropriately.

### Mason jar assay for bees

Adopted from Bahreini et al. [27], glass Mason jars (60 mL, Uline, Canada) (n = 20) were treated with 0.5 mL subsequent dilutions 10,000 mgL^-1^, 1,000 mgL^-1^ or 100 mgL^-1^ of amitraz. Each jar was treated with 3.91 mg, 0.391 mg or 0.0319 mg of amitraz, with four replicates per dilution. Two additional sets of replicates were added: acetone only (solvent control) and no treatment (negative control). The jars were rotated on a hot dog roller as mentioned above, and one sugar cube was hot-glued to the bottom of the jar before 10 newly-emerged bees were added. The top of the jar was covered with a fine mesh screen (16 mesh, Easy Screen®, RCR International Inc., Canada), that was not impregnated with amitraz, and an elastic band was used to secure it around the rim of the jar. The bees were incubated at 33±1°C and 60±5% RH in the dark and bee mortality was assessed 24 h post-treatment. Bees were determined to be dead if they were completely motionless (i.e. no body or appendage movement), and remained motionless when the jar was slightly agitated. The bees and jars were disposed of appropriately.

### The Apiarium assay for mites and bees

For the Apiarium, each side of a plastic strip was coated with 0.25 mL of amitraz dilution using a pipette (0.5 mL in total). Three subsequent dilutions 10,000 mgL^-1^, 1,000 mgL^-1^ and 100 mgL^-1^ of amitraz were tested. Each Apiarium received a plastic strip covered with 3.91 mg, 0.391 mg or 0.0319 mg of amitraz, each with three replicates. Two additional sets of replicates were either treated with acetone only (solvent control) or were not treated (negative control). Apiariums (n = 15) were incubated at 25±1°C and 60±5% RH in the dark for 24 h. After a 4 h exposure, dead mites were counted and removed from the bottom of the container using a fine-tipped paintbrush. Visual assessment of bee mortality was completed by counting the number of dead bees inside the Apiariums. Bees were considered dead if they were immobile, laying on the mesh screen. The Apiarium was gently jostled to ensure there was no response from immobile bees (no body or appendage movement). The Apiariums were then placed in the freezer at -20°C for 2–3 h to kill remaining bees. The total number of bees in the sample were counted and transferred into a 500 mL plastic container to be washed in ethanol (70%) as described by Bahreini et al. [27]. The bees, all parts of Apiariums, and alcohol that contained residues of chemical were disposed of appropriately.

## Statistical analyses

The variables for cumulative mite and bee mortality rates were analyzed using a mixed model (ANOVA) [39]. Amitraz dilutions, solvent and negative controls were main plots. The Apiariums, vials and jars were experimental units and replicates were treated as random effects (PROC MIXED). All treatments were randomly assigned to Apiariums, vials or jars. For the Apiarium assay, the total numbers of mites were calculated by summing all of the mites that dropped during the exposure period and mites that were collected from the bee cluster after the alcohol wash. The cumulative mite mortality was calculated as the proportion of dead mites collected during the exposure time (4 h or 24 h) to the total mites in all replicates of the Apiariums or vials. The proportion of dead bees (cumulative mortality) during the exposure time to the total number of bees was calculated for all replicates of the Apiariums or jars. The normality of the data was determined using the Shapiro-Wilk test (PROC UNIVARIATE). Mortality rates that did not fit a normal distribution were arcsine transformed prior to analyses [40]. The Bonferroni correction was applied to examine differences among treatments (PROC MIXED). The relationship between mite mortality 24 h post-treatment and amitraz dilutions was determined using a fitted polynomial regression model [39].

## Results

### Mite mortality

**Fig 2:**
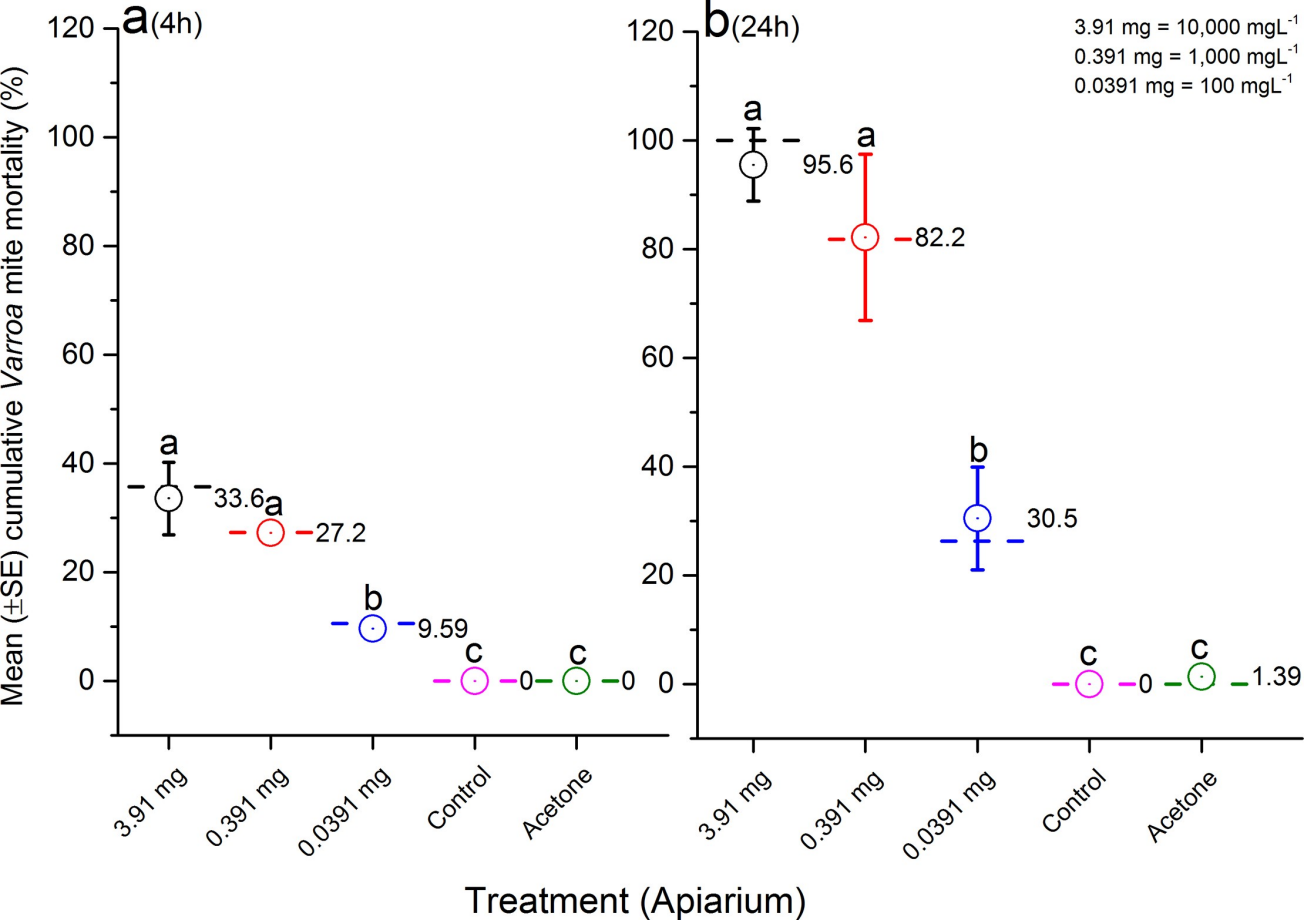
Mean (±SE) cumulative *Varroa* mite mortality (%) in the Apiarium test during 4 h (a) and 24 h (b) assessments. Mites and bees were exposed to different doses of amitraz (3.91 mg, 0.391 mg or 0.0319 mg), solvent (acetone) or left untreated (control). The graph indicates average (open circles and numbers) and median (dash lines) of cumulative mite mortality for replicates in each concentration of amitraz (n = 3), acetone (n = 3) or control (n = 3). Means followed by the same letter among treatments in each assessment are not significantly different (p> 0.05).

Cumulative mite mortality (%) in all treatments of glass vials (34 ± 5.4%) was significantly higher than the Apiarium (13.2 ± 6.1%) after 4 h exposure (F = 6.31; df = 1, 34; p = 0.0154). However, cumulative mite mortality was not significantly different between the vial and Apiarium after 24 h post-treatment (vial: 52.5 ± 9.4% vs Apiarium: 39.4 ± 10.5%) (F = 1.07; df = 1, 34; p = 0.3079). The normality test indicated a normal distribution for the total number of mites in Apiariums (n = 16; p = 0.0532), however, mite mortality was not normally distributed for Apiariums (4h: n = 16; p = 0.0054; 24 h: n = 16; p = 0.0029) or vials (4h: n = 20; p = 0.0003; 24 h: n = 20; p = 0.0017). When *Varroa*-infested bees were exposed to different dilutions of amitraz in the Apiarium, cumulative mite mortality was found to be dose-dependent, and the rate was significantly greater in higher dilutions than lower dilutions after 4 h exposure (F = 208.45; df = 4, 11; p< 0.0001) (Fig 2A) and 24 h post-treatment (F = 90.15; df = 4, 11; p< 0.0001) (Fig 2B). In other words, high *Varroa* mite mortality (≥82%) was recorded for higher doses (3.91 mg and 0.391 mg) of amitraz in the Apiarium and vial during a 24 h trial.

**Fig 3:**
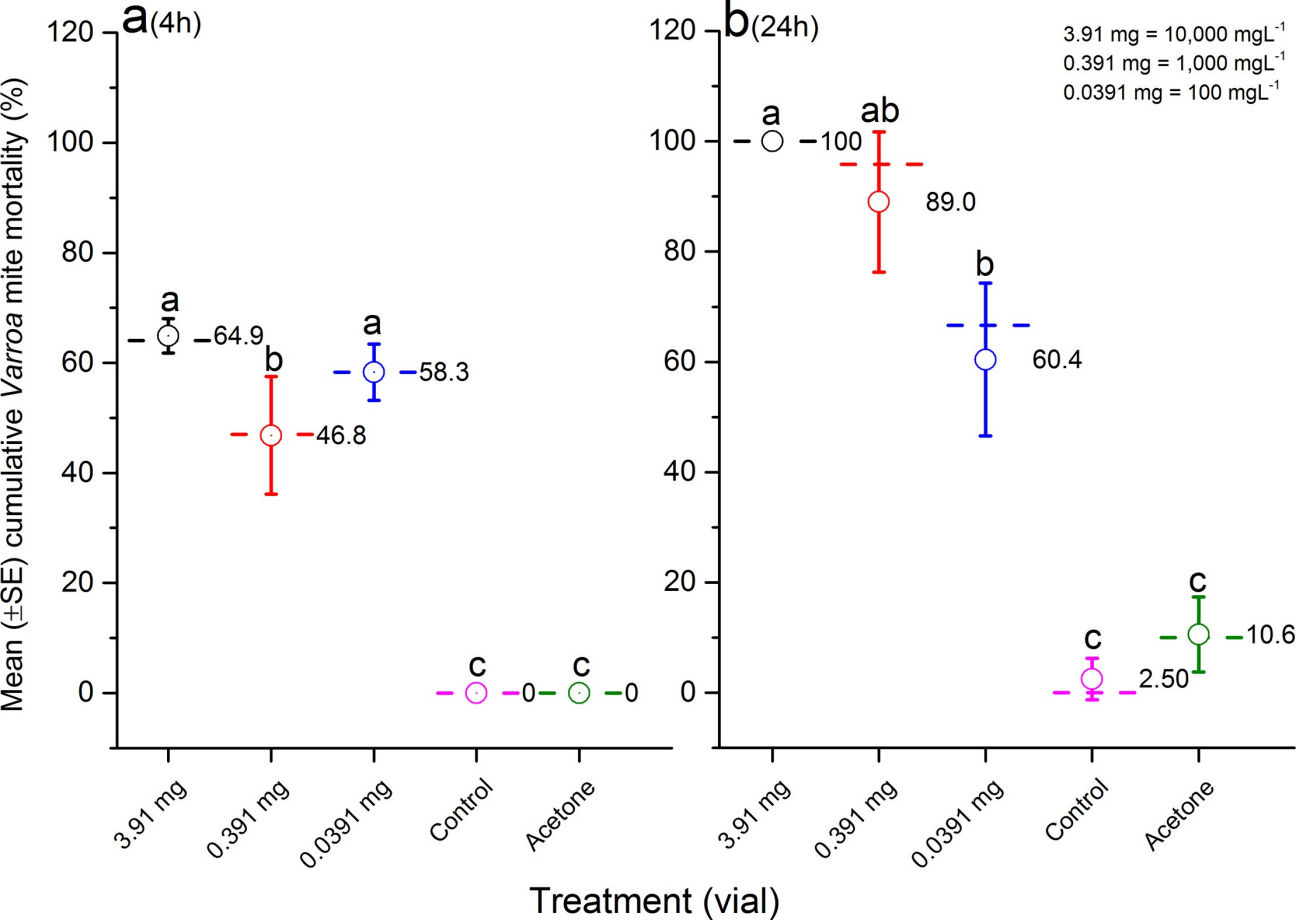
Mean (±SE) cumulative *Varroa* mite mortality (%) in the glass vial test during 4 h (a) and 24 h (b) assessments. Mites and bees were exposed to different doses of amitraz (3.91 mg, 0.391 mg or 0.0319 mg), solvent (acetone) or left untreated (control). The graph indicates average (open circles and numbers) and median (dash lines) of cumulative mite mortality for replicates in each concentration of amitraz (n = 3), acetone (n = 3) or control (n = 3). Means followed by the same letter among treatments in each assessment are not significantly different (p> 0.05).

In glass vials where mites were in direct contact with amitraz, the mite mortality was not dose-dependent at 4 h exposure (F = 246.3; df = 4, 15; p< 0.0001) (Fig 3A), but mite mortality was dose-dependent at 24 h post-treatment (F = 37.98; df = 4, 15; p< 0.0001) (Fig 3B). For the first 4 h exposure, amitraz killed double the number of mites in direct contact with the active ingredient in the glass vial (≤ 64%) compared to indirect contact in the Apiarium (≤ 34%). Cumulative mite mortality rates in the glass vial and Apiarium were similar at the end of trials (24 h assessment). The solvent treatment (acetone) and negative control had a significantly lower mite mortality rate than all dilutions of amitraz (p< 0.05).

**Fig 4:**
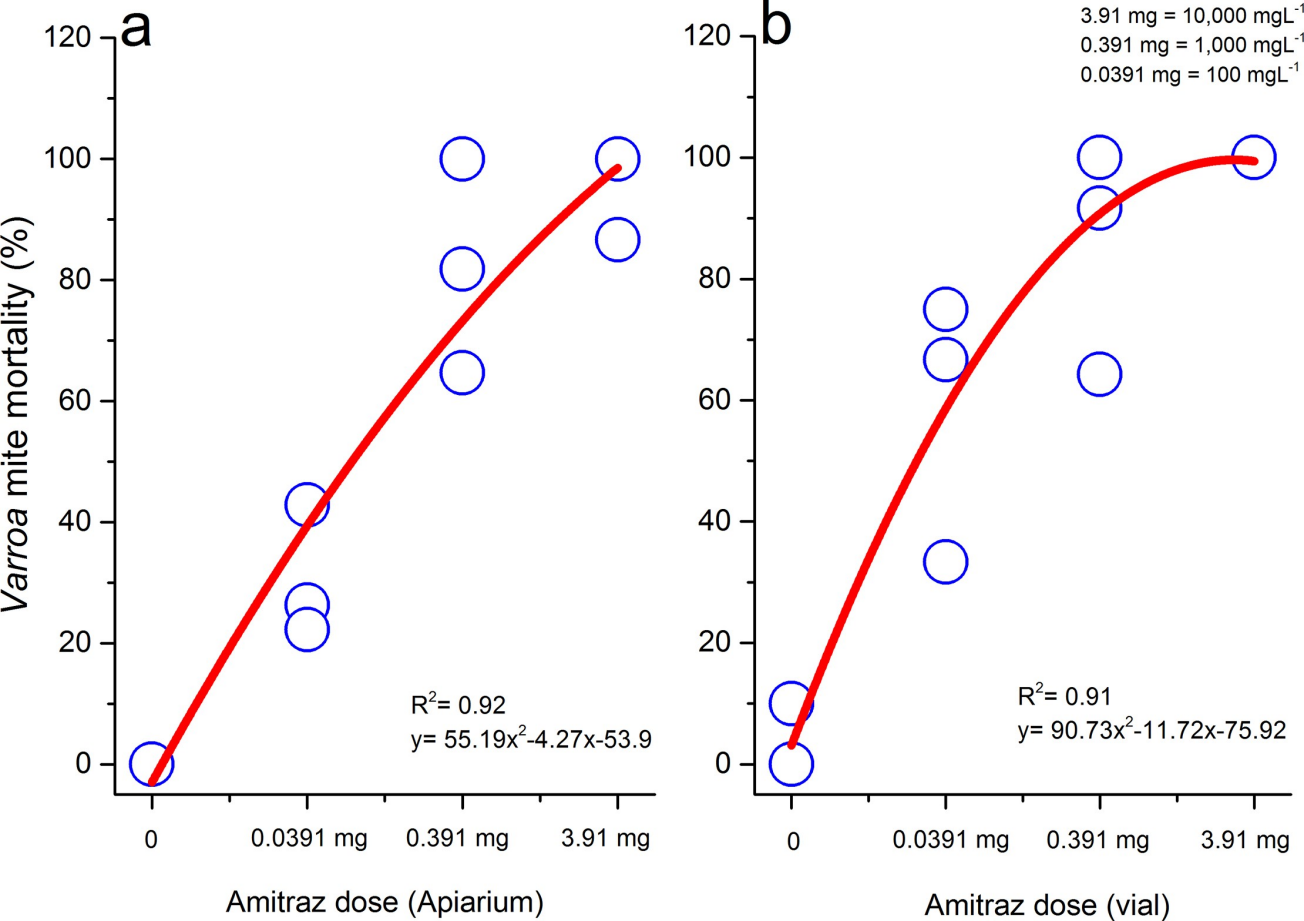
Effect of the dose rates of amitraz (3.91 mg, 0.391 mg and 0.0319 mg) on *Varroa* mite mortality 24 h post-treatment in the Apiarium (a) and vial (b) trials. Each open circle represents the mean value of mite mortality (%) for each amitraz dilution.

A fitted polynomial function was applied to determine the amitraz dose-mite mortality relationship. Results indicted a significant polynomial relationship between *Varroa* mite mortality during 24 h post-treatment with dose rates of amitraz for Apiarium (df = 12; p = 0.029) (Fig 4A), and vial (df = 13; p = 0.0007) trials (Fig 4B).

### Bee mortality

No dead bees were found in the Apiarium or Mason jar after 4 h of exposure, but cumulative bee mortality (%) in the Mason jar (49.5 ± 7.5%) was significantly higher than the Apiarium (1.8 ± 8.4%) 24 h post-treatment (F = 17.89; df = 1, 34; p = 0.0002). A non-significant and low rate of bee mortality (< 3%) was observed during 24 h trials across all treatments in the Apiarium (F = 1.99; df = 4, 11; p = 0.1663). The Shapiro-Wilk test indicated a normal distribution among the total number of bees in Apiariums (n = 16; p = 0.4875); however, the results of 24 h bee mortality was normally distributed only for Apiariums (n = 16; p = 0. 6589), not for jars (n = 20; p = 0.0003). Bee mortality was dose-dependent in the Mason jar, where higher dilutions of amitraz killed significantly more bees (> 40%) when compared to controls during 24 h post-treatment (F = 58.97; df = 4, 15; p< 0.0001). The solvent control treatment had low mortality for bees (< 4.4%), similar to the negative control (< 2.5%) in both the Mason jar and Apiarium (Table 1).

**Table 1:** Cumulative bee mortality (%) in treatments.

Container	Duration (h)	Acetone 99.9%	Amitraz 0.0391 mg	Amitraz 0.391 mg	Amitraz 3.91 mg	Control
Apiarium	4	0^a^	0^a^	0^a^	0^a^	0^a^
	24	1.25±0.54^a^	2.91±0.62^a^	1.76±0.62^a^	2.71±0.62^a^	0.84±0.62^a^
Mason jar	4	0^a^	0^a^	0^a^	0^a^	0^a^
	24	4.36±3.74^c^	40.68±3.74^b^	100±3.74^a^	100±3.74^a^	2.5±3.74^c^

Mean (±SE) cumulative bee mortality (%) in treatments when worker bees were exposed to different doses of amitraz (3.91 mg, 0.391 mg or 0.0319 mg), solvent (acetone) or left untreated (control) in a 800 mL Apiarium or in a 60 mL Mason jar through 4 h and 24 h assessments. Each value represents an average of replicates for jars (n = 5) or Apiariums (n = 3). Means within each row followed by the same letter among treatments are not significantly different (p> 0.05).

## Discussion

*Varroa* mites are a lethal ectoparasite of the honey bee and have been recognized as a main cause for winter colony mortality in Canada [41]. Since resistance to Varroacides has been reported multiple times in North America over the last two decades [11, 13, 28], it is necessary to find novel miticides with different modes of action for *Varroa* control. As historical bioassay methodologies have generally tested chemicals on bees and mites separately, there is an urgent need for a bridge between laboratory and field experiments. Using amitraz as a reference chemical, our findings suggest that the newly-designed Apiarium method could provide intermediary testing after laboratory bioassays and before moving to field tests. The Apiarium can be an essential component of screening new chemicals because it simulates a colony-like environment where mites and bees are exposed to chemicals simultaneously. Additionally, it allows researchers to test different delivery methods for new chemical products.

Researchers have developed many bioassay cages using a variety of sizes, shapes, and materials to measure the lethal toxicity and efficacy of chemicals against *Varroa*. Previous studies have used Petri dishes [14, 42–44], glass vials [27–29, 45], polypropylene vials [33], capsules [30], impregnated wax [31], topical application methods [15, 27], glass Mason jars [27, 38] or plastic containers [13]. Of these different bioassays, Elzen et al. [45] and Kamler et al. [33] suggested that the glass or polypropylene vial methods be used for evaluating resistance of *Varroa* to miticides. For this reason, the glass vial was chosen to compare to our newly developed Apiarium for miticide efficacy. Vandervalk [29] was the first to screen five commercial synthetic miticides, not registered for use in honey bee colonies in Canada, on *Varroa* using the glass vial method. The study found the highest mite mortality rates for formulated products containing clofentezine, spiromesifen, bifenazate, and acequinocyl at a 100,000 mgL^-1^ dilution. Vandervalk’s [29] results demonstrated that the mite mortality rate gradually decreased with lower dilutions in a dose-dependent manner. Not only were we able to repeat dose-dependence in both the vial and Apiarium assays after 24 h post-treatment, our results confirm that the Apiarium was comparable to the vial test in mite mortality assessment. Similar to Almecija et al. [17], we found a relationship of dose-dependency between amitraz dilution and mite mortality in susceptible mite populations. Achieving dose-dependency, in addition to high mite mortality, is a promising metric for using the Apiarium to evaluate toxicity values (i.e. LC_50_) for simultaneous exposure of bees and mites in the same environment. Toxicity values are a scientific standard for chemical screening and are important for future programs to develop dose-response curves. Ultimately, dose-response curves are the key factor to finding the optimum field dose of a chemical that is safe for honey bees and lethal for *Varroa* mites.

Conventional laboratory bioassays are an essential component of initial screening for new miticides and determining lethal toxicity. The challenge is reproducing the same results in the field obtained under laboratory conditions. This was seen in a number of studies on neonicotinoids where conflicting results between the laboratory and field tests highlighted additional colony mechanisms that are not accounted for when screening chemicals in the lab [46, 47]. The Apiarium was designed to simulate a colony-like environment to capture some of these interacting mechanisms and provide a more realistic picture of miticide efficacy. Although this is a preliminary study, our results show differences between direct and indirect contact for mite mortality when comparing the Apiarium and glass vial methods. Despite non-significant mite mortality between the Apiariums and vials after 24 h post-treatment with amitraz, mite mortality after 4 h was almost double in the vials. The high mite mortality in vials after 4 h is most likely associated with continuous exposure to the compound on the inner surface of the vial. Although the exclusive use of the vial bioassay test as a tool for evaluating a chemical seems promising, it may not translate to represent mite mortality in bee colonies used in field trials. In colonies, *Varroa*-infested bees are freely moving in the cluster and are not necessarily in direct contact with the applied miticide at all times. The Apiarium, for this reason, provides a better assessment of how contact miticides interact with mites in a colony. Another factor to consider is the number of mites present in the infested bee cluster when testing in the Apiarium. Insufficient parasite numbers can lead to an overrepresentation of mite mortality and misleading results for miticide efficacy [38]. For example, if there are only five mites in the sample, the death of one mite equates to 20 percent mortality. Therefore, having enough mites in the sample and adjusting mean abundance of *Varroa* in the bee cluster is recommended for the Apiarium bioassay tests.

The Apiarium produced substantially different results for honey bee mortality when compared to the Mason jar method. Bee mortality was 100% at higher doses of amitraz (3.91 mg and 0.391 mg) in the Mason jars, but averaged 2% mortality for all doses in the Apiarium. With this in mind, the Apiarium appears to be capturing some of the colony mechanisms, giving insight to how a miticide might function in a colony environment. It is important to understand how the interaction between the compounds and total number of mites in each replicate could affect honey bee survival. A higher density of parasites may lead to host mortality through synergistic interaction with stressors such as lack of queen and brood pheromones, inability to fly and defecate, chemical exposure, and confinement. These stressors affect host physiology, behavior and longevity [48–50]. In our Apiarium assay, honey bee mortality was low in control groups (≤0.84%) when mean abundance of *Varroa* averaged 11% and the number of bees in the samples averaged 135 bees (60 mL of bees). At this level of infestation, V*arroa* were not a contributing factor to honey bee mortality over the 24 h testing period. We suggest adjusting the number of bees and mites in the Apiarium to develop a threshold for maximum mite infestations before bee survival is impaired. The use of a non-treated control in the experiment is also important to ensure that bees survived under the tested conditions and that no other stress factors are compromising the results. These two factors are particularly important for longer duration experiments, where stressors may need to be controlled to eliminate confounding factors.

There are other factors that could have influenced the bee mortality results. The differences in honey bee ages between methodologies may have affected the results as bees are more susceptible to active ingredients at early life stages [51]. The justification for using newly emerged bees in the Mason jars was to be consistent with our previous investigation and to adhere to standard protocols for screening miticides in the laboratory [27]. Mix-aged bees were used in the Apiarium to replicate bee population demographics with natural mite infestation similar to a colony environment. Based on the presented results, incorporating the Apiarium as a part of conventional miticide screening will ensure we are providing conditions similar to a honey bee colony. In addition, screening new chemicals without including the Apiarium bioassay could potentially over or underestimate a miticides’ toxicity. For instance, if amitraz was a new chemical being screened for miticide efficacy and toxicity to bees using the Mason jar bioassay, it would likely be removed from further testing due to unacceptable bee mortality. This is a good example as toxicity of amitraz to honey bees has been reported [52–54], yet it has been used safely and successfully in honey bee colonies for more than a decade.

Our study shows the Apiarium bioassay can provide a bridge between testing in the laboratory and the field to reduce factors that may cause discrepancies in results. Another benefit of using the Apiarium is the ability to test application methods for a new miticide. This is an important step as the parasitic mite-miticide interaction is complicated by *Varroa* behavior in a colony. For instance, *Varroa* are most vulnerable to contact-based miticides when they are on adult bees in the phoretic phase [55]. Ramsay et al. [56] found that > 80% of *Varroa* outside brood cells were located under the ventricle abdominal segment of honey bees. These sternites could be providing a protective shield from bee grooming behavior [57] and likely inhibit direct contact with miticides. This behavior needs to be taken into consideration when testing different application methods before field trials because it can affect the efficacy of the treatment. The Apiarium method can be easily modified by changing the bottom of the 1000 mL container to address desired application needs (e.g. strip, oral feeding for systemic pesticides, gels, etc.). Using the Apiarium method before field application will help select compounds that are the most compatible when bees and mites are in a cluster and simultaneously exposed to the tested chemical.

Once a successful compound is selected from laboratory trials, it must be evaluated under field conditions. There is considerable investment in resources when scaling up to a field test. The cost of bees, equipment, materials, chemicals, and personnel may be a barrier to conducting field research. Not only that, after experiment completion, the equipment and bees must be safely disposed. This is required to prevent contamination in the surrounding environment and other colonies via drifting and robbing. These factors may add a financial burden as researchers must procure new equipment and honey bees each time the chemicals are tested. This exemplifies the importance of using the Apiarium as an intermediate method to test miticides. This can save a considerable amount of time and resources, and ensure field programs run more efficiently by only screening the most effective chemicals.

In this study, we developed a new cage and a bioassay method for screening novel miticides. The purpose of designing the Apiarium was to complement, not replace, existing conventional methods before moving to field trials. The results of this study conclude the Apiarium provides an inexpensive, rapid, simple, and reliable method for screening potential miticides in the laboratory. Using the Apiarium bioassay, where bees and mites are simultaneously exposed to chemicals under conditions similar to honey bee colonies, provides an environment to determine other metrics such as: lethal doses, toxicity ratio, selective ratio, resistance ratio, and acute/chronic contact or oral toxicity (gravity feeders installed). With modification, this Apiarium could also be used to test other miticides such as organic acids or essential oils, or provide a tool for measuring the degree of resistance in *Varroa* populations. There is potential, with modification, that the Apiarium could be used when developing a new resistance-testing standard. This method could also be applied to examine the effects of pesticides on other bee endoparasites and ectoparasites such as *Acarapis woodi* (Rennie), *Tropilaelaps* spp., and *Braula coeca* Nitzsch. For studies using similar methods for testing novel chemicals on honey bees and *Varroa* mites, we suggest examining and standardizing a few metrics more thoroughly: area (length and width) of application strips, chemical dose, exposure times, sizes of bee clusters, and mean abundance of *Varroa* mites. Overall, the Apiarium provides a versatile method to reliably test many different metrics on honey bees and their associated pests, providing confidence when transitioning from laboratory to field experimentation.
